# Impact of CFTR modulators on exercise capacity in adolescents with cystic fibrosis

**DOI:** 10.1183/23120541.00687-2023

**Published:** 2024-02-19

**Authors:** Molla Imaduddin Ahmed, Naomi Dayman, Natalie Blyth, Joe Madge, Erol Gaillard

**Affiliations:** 1 Department of Paediatric Respiratory Medicine, University Hospitals of Leicester NHS Trust, Leicester, UK; 2 Department of Respiratory sciences, University of Leicester, Leicester, UK

## Abstract

**Background:**

Exercise capacity is an independent predictor of clinical worsening in cystic fibrosis (CF). There is limited evidence of the impact of cystic fibrosis transmembrane conductance regulator (CFTR) modulators on exercise capacity in children with CF. The aim of the present study was to assess the impact of CFTR modulators on exercise capacity in a cohort of adolescents with CF.

**Methods:**

A prospective single-centre cohort study was carried out. Cardiopulmonary exercise testing (CPET) was performed at baseline, prior to starting Symkevi or Kaftrio and between 4 and 8 months after starting treatment.

**Results:**

19 adolescents with CF had CPET performed prior to and after CFTR modulator treatment, between December 2019 and March 2022. Breathing reserve improved in the whole cohort, with greater improvement in the modulator-naïve patients after starting treatment with Kaftrio. There was no improvement in peak oxygen uptake and anaerobic threshold after 4 to 8 months of treatment with CFTR modulators.

**Conclusion:**

Exercise testing with CPET can be used as an additional tool to monitor response to CFTR modulators. Breathing reserve on CPET may provide a surrogate marker to monitor the improvement in CF lung disease with CFTR modulator treatment.

## Shareable abstract

**Exercise testing with CPET can be used as an additional tool to monitor response to CFTR modulators; improvement in breathing reserve was noted in the whole cohort and was significant for modulator-naïve children started on elexacaftor/tezacaftor/ivacaftor** https://bit.ly/47oQHzc

## Introduction

Cystic fibrosis (CF) is one of the most common inherited conditions, which causes abnormalities in the cystic fibrosis transmembrane conductance regulator (CFTR) gene [1]. This results in a multisystem disease with progressive lung disease being its main feature. Patients with CF have varying degrees of exertional breathlessness with subsequent impact on their exercise capacity. Exercise capacity has been noted to be an independent predictor of clinical worsening in CF [2, 3]. Owing to underlying lung abnormalities, and increased dead space in their lungs, patients with CF have ventilatory limitation, which limits their exercise capacity [4]. Physical deconditioning in children with CF can also cause reduced exercise capacity [5]; this may be due to multiple factors including parental overprotection and reduced understanding of the disease [6]. Exercise training programmes have been shown to help improve exercise capacity in CF patients [7] and, thereby, have a positive impact on overall lung health.

Cardiopulmonary exercise testing (CPET) is considered the gold standard investigation to test exercise capacity. CPET parameters correlate with radiological deterioration [8] in CF patients as well as worsening of the ventilation homogeneity [9]. Exercise testing in CF patients therefore offers additional information that is helpful to not only detect early evidence of lung disease [10] and ascertain the progression of lung disease, but also monitor for effects of new treatments. CPET has been suggested, in addition to lung clearance index, as an outcome measure for clinical trials [11]. CPET needs specialist equipment and staff expertise, and is therefore not widely available for CF patients. The European Respiratory Society (ERS) statement on CPET highlights the importance of having a methodical and standardised approach for the assessment of the CPET parameters to determine the cause of exercise limitation [12]. Regular evaluation of exercise capacity also helps in supporting the CF patients in developing an individualised exercise programme [13] that not only has a positive impact on their lung disease [14], but also on their overall quality of life [15].

New CFTR modulators have significantly improved CF outcomes in the last few years. The treatment with CFTR modulators is associated with improvements in lung function and nutritional status [16–20]. In CF patients homozygous for the F508del mutation, use of Orkambi (lumacaftor/ivacaftor) and Symkevi (tezacaftor/ivacaftor) result in an absolute increase in forced expiratory volume in 1 s (FEV_1_) % predicted by 4 percentage points [16, 17]. In clinical trials, the recently introduced CFTR modulator Kaftrio (elexacaftor/tezacaftor/ivacaftor) has been shown to improve FEV_1_ on average by >10% [18, 19].

Symkevi and Kaftrio are now available for children aged 12 years and above in the UK [21]. There is however limited evidence of the impact of CFTR modulators on exercise capacity in CF children. A recent study of three CF adolescents on Kaftrio showed improvement in peak oxygen uptake (*V*′_O_2__) after 6 weeks of Kaftrio treatment [22]. A recent Danish study involving 91 patients showed statistically significant improvement in peak *V*′_O_2__ and maximal workload on CPET 12 months after initiation of treatment with Orkambi or Symkevi [23]. Other previous studies have reviewed the impact of Orkambi or Ivacaftor on exercise capacity in CF patients; two of these studies did not show any significant change in exercise capacity with these treatments [24–26]. The aim of our study was to assess the impact of CFTR modulators on exercise capacity in our cohort of CF adolescents.

## Methods

### Study design

A prospective observational cohort study was carried out.

### Participants

All adolescents (12–18 years old) under follow-up at our paediatric CF centre, who were eligible for treatment with CFTR modulators following licensing approval for these medications in the UK, were eligible for inclusion in the study. These adolescents had their CF diagnosed based on the presence of two disease-causing CFTR mutations and/or a sweat chloride ≥60 mmol·L^−1^. Exclusion criteria included episodes of infective respiratory exacerbations within the previous month, treatment with antibiotics other than prophylactic antibiotics, systemic corticosteroids or initiation of any other chronic new treatments within the previous month.

All aspects of this study were approved by the East Midlands Research Ethics Committee (reference number 12/WM/0285). Written informed consent was obtained from parents or legal guardians.

### Pulmonary function tests

All the participants performed spirometry following the American Thoracic Society (ATS)/ERS (2019) guidelines [27], and nutritional assessment (height, weight and body mass index (BMI)) was carried out at baseline, prior to starting Symkevi or Kaftrio and 4–8 months after starting treatment.

### Cardiopulmonary exercise testing

All participants had CPET performed as per the current Association for Respiratory Technology and Physiology (ARTP) recommendations [28]. The CPET was performed on a cycle ergometer (Ergoselect 100, Ergoline, Bitz, Germany). A maximal ramp-incremental protocol was followed [29]. Following a 3-min rest period, there was 1 min of unloaded cycling, at which point the ramp protocol began where workload increased at a predetermined rate (15–20 W·min^−1^). The level of the ramp in exercise was determined by the individual's current fitness/exercise levels to ensure target test duration of 10±2 min was achieved. Whilst exercising, the patients were asked to maintain a speed of 60 rpm and continue at this rate until they could no longer maintain this rate despite encouragement. At the stage when the patient was no longer able to continue at the set speed, testing was discontinued, resistance was reduced to 10 W and patients were encouraged to continue cycling for 3–5 min. Heart rate and electrocardiograph were monitored using the integrated ECG (Medicard, Medisoft, Sorinnes, Belgium). Pulse oximetry was performed on a beat-to-beat basis to ascertain saturations (Nonin 8000S, Nonin Medical, Tilburg, The Netherlands). Blood pressure was performed at 2-min intervals using the integrated device (Ergoselect, Ergoline, Bits, Germany). Patients were tested at the predetermined wattage increase as set out on the baseline testing.

CPET parameters recorded included peak *V*′_O_2__, volume of exhaled carbon dioxide (*V*′_CO_2__), minute ventilation (*V*′_E_), respiratory exchange ratio (*V*′_CO_2__/*V*′_O_2__) and ventilatory anaerobic threshold (VAT). Breathing reserve was calculated as a ratio of peak *V*′_E_ to maximum voluntary ventilation (MVV). MVV was calculated as FEV_1_ × 40 [12].

### Statistical analysis

Statistical analysis was performed using SPSS Statistics 28.0.1. Data on clinical and CPET parameters were presented as mean±sd. We performed Wilcoxon signed rank test to review the differences in parameters following treatment with CFTR modulators. A p-value <0.05 was considered as statistically significant.

## Results

54 cardiopulmonary exercise tests were performed between December 2019 and March 2022. Mean±sd age of the study cohort at the beginning of the study was 15.2±2.1 years.

19 adolescents (six males) underwent CPET at baseline and after modulator initiation. 11 adolescents were studied before and after treatment with Symkevi; nine of them were subsequently switched to Kaftrio. Eight adolescents were started on Kaftrio as their first CFTR modulator. Two patients had no follow-up CPET, one due to anxiety and the other because they chose not to start the modulator treatment.

Exertional breathlessness was the limiting factor in one CPET study, and this was noted at baseline prior to treatment with the CFTR modulator; lower limb fatigue was the reason for stopping in the other 53 CPET studies.

Table 1 shows the difference in clinical and CPET parameters at baseline and at the end of the study after being on treatment with CFTR modulators (Symkevi or Kaftrio).

**TABLE 1:** Clinical and cardiopulmonary exercise testing parameters for the study cohort (n=19)

	**Pre-modulator**	**Post-modulator**	**p-value**
**FEV_1_ % predicted**	86±12.3	94.7±11.3	**0** **.** **034**
**BMI kg·m^−2^**	19.9±3.2	20.7±3.5	0.445
**Peak *V*′_O_2__ mL·min^−1^·kg^−1^**	33.1±6.7	30.4±7.9	0.719
**VAT % of peak *V*′_O_2__**	47.6±15.4	52.9±12.7	0.234
**BR %**	30.3±12.3	43.7±14.4	**0** **.** **004**
**Maximal workload W**	75.2±13.4	72.7±13.5	0.941

Data are presented as mean±sd. p-values <0.05 are highlighted in bold. FEV_1_: forced expiratory volume in 1 s; BMI: body mass index; *V*′_O_2__: oxygen uptake; VAT: ventilatory anaerobic threshold; BR: breathing reserve.

### CPET after initiation of Symkevi treatment

11 patients with a mean age of 15.1±2.9 years were started on Symkevi treatment; three of them were males. 10 of these patients were homozygous for DF508 and one patient was heterozygous for 711+3A>G. These patients had follow-up CPET performed after 6–8 months of Symkevi treatment. Table 2 shows the difference in clinical and CPET parameters following treatment with Symkevi.

**TABLE 2:** Clinical and cardiopulmonary exercise testing parameters for patients who started on Symkevi (n=11)

	**Baseline**	**Symkevi**	**p-value**
**FEV_1_ % predicted**	87.3±14.6	91.1±15.9	**0** **.** **036**
**BMI kg·m^−2^**	20.4±2.5	20.8±2.6	0.212
**Peak *V*′_O_2__ mL·min^−1^·kg^−1^**	33.5±8.3	34.6±8.8	0.929
**VAT % of peak *V*′_O_2__**	40.5±11.4	49.9±14.1	0.126
**BR %**	26.7±12.1	32.1±17.9	0.213
**Maximal workload W**	71.4±11.2	68.9±11.3	0.261

Data are presented as mean±sd. p-values <0.05 are highlighted in bold. FEV_1_: forced expiratory volume in 1 s; BMI: body mass index; *V*′_O_2__: oxygen uptake; VAT: ventilatory anaerobic threshold; BR: breathing reserve.

Of the 11 patients on Symkevi, nine switched to Kaftrio after being on Symkevi for 6–8 months. One child was transitioned to an out of area adult CF centre and another child who was heterozygous for 711+3A>G continued on Symkevi.

### Kaftrio treatment

Kaftrio was started in 17 of our CF patients (including nine that were previously on Symkevi) who were between 12 and 18 years old. Eight patients started on Kaftrio were not previously on any CFTR modulators. Repeat clinical assessment and CPET was performed 4–6 months after starting Kaftrio treatment.

#### Patients switched from Symkevi

Nine children (five males) were started on Kaftrio following 6–8 months of treatment with Symkevi. Mean age was 16±1.9 years at the initiation of Kaftrio. All these patients were homozygous for DF508. Table 3 highlights the changes in clinical and CPET parameters for patients switched from Symkevi to Kaftrio.

**TABLE 3:** Clinical and cardiopulmonary exercise testing parameters for patients switched from Symkevi to Kaftrio (n=9)

	**Post-Symkevi**	**Post-Kaftrio**	**p-value**
**FEV_1_ % predicted**	93.2±15.1	97.9±11.4	0.161
**BMI kg·m^−2^**	20.8±2.9	21.2±2.7	0.440
**Peak *V*′_O_2__ mL·min^−1^·kg^−1^**	36.3±8.9	33.9±6.4	0.326
**VAT % of peak *V*′_O_2__**	52±12.3	41.9±14.8	0.236
**BR %**	30.9±19.3	36.1±14.6	0.441
**Maximal workload W**	69.1±12.5	67.7±11.7	0.779

Data are presented as mean±sd. FEV_1_: forced expiratory volume in 1 s; BMI: body mass index; *V*′_O_2__: oxygen uptake; VAT: ventilatory anaerobic threshold; BR: breathing reserve.

#### Patients with Kaftrio as their first CFTR modulator

Eight adolescents were started on Kaftrio as their first CFTR modulator, one of whom was male. Four of them were homozygous for DF508 and the remaining four were heterozygous for DF508 and minimal function mutation. Table 4 shows the difference in clinical and CPET parameters for modulator-naive patients at baseline and at the end of the study after being on treatment with Kaftrio.

**TABLE 4:** Clinical and cardiopulmonary exercise testing parameters for modulator-naïve patients started on Kaftrio (n=8)

	**Pre-Kaftrio**	**Post-Kaftrio**	**p-value**
**FEV_1_ % predicted**	80.2±7.2	90.3±5.7	**0** **.** **046**
**BMI kg·m^−2^**	19±5.2	19.3±4.9	0.416
**Peak *V*′_O_2__ mL·min^−1^·kg^−1^**	33.4±6.1	32.4±8.4	0.6
**VAT % of peak *V*′_O_2__**	49±17.7	54±11.2	0.249
**BR %**	25.8±7.2	41.7±10.0	**0** **.** **028**
**Maximal workload W**	83.4±18.8	77.8±9.5	0.528

Data are presented as mean±sd. p-values <0.05 are highlighted in bold. FEV_1_: forced expiratory volume in 1 s; BMI: body mass index; *V*′_O_2__: oxygen uptake; VAT: ventilatory anaerobic threshold; BR: breathing reserve.

## Discussion

We assessed the impact of CFTR modulators on exercise capacity and found improvements in breathing reserve in the whole cohort, but this was only significant for the modulator-naïve children started on Kaftrio. In children who were initially treated with Symkevi and then switched to Kaftrio, there were additional small improvements noted in most parameters, but these did not reach statistical significance likely due to small patient numbers. Associated significant improvements in the FEV_1_ % predicted were also noted.

Improved exercise capacity has a positive impact on the overall health of CF patients [4] and is related to overall improved survival [3]. CPET is the gold standard testing for exercise capacity, and international recommendations are to perform CPET at least once every year in CF patients [30]. CPET is infrequently performed in the paediatric age group due to poor access to equipment and expertise in exercise testing. We performed CPET in our patients prior to initiation of CFTR modulators and thereafter for monitoring of exercise capacity, we obtained reliable results in our cohort of CF adolescents.

There are limited published data on impact on exercise tolerance following the use of CFTR modulators, particularly Kaftrio. A recent study involving nine CF adults with severe lung disease showed improvement in respiratory muscle strength and improved exercise capacity assessed by the 6-minute walk test following Kaftrio treatment [31]. A study from Causer *et al.* [22] showed improvements in peak *V*′_O_2__, maximal workload and the anaerobic threshold with 6 weeks of Kaftrio treatment. Rysgaard *et al.* [23] performed a study involving 91 CF patients 12 years of age and older who were started on Orkambi and Symkevi, and showed significant improvements in peak *V*′_O_2__ and maximal workload on CPET after 1 year of treatment. A smaller study reviewed the long-term impact of Orkambi on exercise capacity in three CF adult patients and reported improvement in exercise capacity and daytime activity levels following 2 years of treatment. All three patients had an improvement in their peak *V*′_O_2__ and VAT [24]. A previous study by Wilson *et al.* [25] studied the impact of Orkambi on exercise tolerance in CF patients compared to placebo treatment; the authors reported no improvement in CPET parameters for patients on Orkambi compared to those on placebo. Another study involving 11 CF adults also showed that treatment with Orkambi did not have any significant impact on the exercise time, CPET parameters including peak *V*′_O_2__, exercise-induced breathlessness or leg discomfort following exercise [32]; however, the authors studied the patients after only 1 month of treatment. Edgeworth and colleagues [26] published the results of a double-blind, placebo-controlled crossover study in 2017 involving 20 CF patients with the G551D mutation, and reported that treatment with ivacaftor increased mean exercise time on CPET but with no associated improvement in *V*′_E_ or *V*′_O_2__ max. We found improvements in breathing reserve following treatment with CFTR modulators, but this has not been reported in any previous studies. However, as noted in the previous studies, we did not find any significant improvement in peak *V*′_O_2__ and VAT following treatment with CFTR modulators.

A low breathing reserve (<15%) [12] noted on CPET suggests a respiratory pathology, particularly when associated with low peak *V*′_O_2__. Progressive lung disease in CF causes ventilatory limitation on exercise capacity, and there is also an increase in dead space that correlates with worsening lung disease [4]. The improvement in breathing reserve was significant in our modulator-naïve patients after starting on Kaftrio, as well as in the overall cohort. Breathing reserve may therefore provide a surrogate marker to monitor the improvement in CF lung disease with CFTR modulator treatment.

*V*′_O_2__ is the most important CPET parameter, and a normal peak *V*′_O_2__ rules out any significant cardiorespiratory pathology for reduced exercise capacity. A recent systematic review established a correlation between peak *V*′_O_2__ and risk of death in CF patients and found that low peak *V*′_O_2__ increases the risk of death in CF patients nearly fivefold [33]. A study by Kampouras *et al.* [34] showed that patients with peak *V*′_O_2__ <60% have an increased risk of subsequent respiratory exacerbation and worsening of their lung health. Peak *V*′_O_2__ values in normal adolescent boys are in the range of 48–50 mL·kg^−1^·min^−1^ and in adolescent girls 35–45 mL·kg^−1^·min^−1^ [35]. Patients with CF show a progressive reduction in their *V*′_O_2__ values associated with worsening of their lung disease [36]. In our cohort of patients, we did not find significant improvement in peak *V*′_O_2__ with CFTR modulator treatment, and this was similar to the findings of most previous studies.

Studies have shown that the maximal exercise capacity in CF can improve with exercise programmes [37], and VAT can be used to assess this improvement. An improvement in VAT suggests improved capacity for exercise at higher intensities for longer periods. This not only improves the lung health of CF patients, but also has a positive impact on their quality of life [15]. This highlights the influence of reduced activity levels on VAT resulting in physical deconditioning [38], and this may be a factor in our cohort as their activity levels were reduced due to COVID-19 related shielding and lockdowns. In our cohort of CF patients, there was a trend towards improving VAT, but this was not statistically significant.

Improvement in lung function with Kaftrio is well described [18, 19]. We noted improvement in FEV_1_ with CFTR modulator treatment in our cohort of CF patients which was consistent with previous studies.

Studies have shown that CFTR modulators are associated with improvement in BMI [39]. This may be related to improvements in exercise capacity; however, there is a lack of evidence to substantiate this. In our patient cohort, BMI improved but did not reach significance.

One limitation of our study is the relatively small number of patients involved. The follow-up CPET studies were initially planned 4–6 months after initiation of treatment with CFTR modulators, but some delays occurred due to the COVID-19 pandemic. It is unclear whether this delay had any impact on the results for our cohort of CF patients.

### Conclusion

We have demonstrated that exercise testing with CPET can be used as an additional tool to monitor response to CFTR modulators. Although we did not note significant changes in peak *V*′_O_2__ and VAT, this is the first published report of CPET in CF patients on CFTR modulators where breathing reserve has shown significant improvement. Longitudinal studies are required to ascertain the impact of CFTR modulators on cardiopulmonary fitness in CF patients.
